# Profile of the Gut Microbiome Containing Carbapenem-Resistant Enterobacteriaceae in ICU Patients

**DOI:** 10.3390/microorganisms10071309

**Published:** 2022-06-28

**Authors:** Anees A. Sindi, Sarah M. Alsayed, Ibrahim Abushoshah, Diyaa H. Bokhary, Nisreen R. Tashkandy

**Affiliations:** 1 Department of Anesthesia and Critical Care, Faculty of Medicine, King AbdulAziz University, Jeddah 21598, Saudi Arabia; asindi2@kau.edu.sa (A.A.S.); iabushoshah@kau.edu.sa (I.A.); 2 Department of Biological Sciences, Faculty of Science, King AbdulAziz University, Jeddah 21598, Saudi Arabia; sabdulazizalsayed@stu.kau.edu.sa; 3 Department of Emergency Medicine, King Abdulaziz University Hospital, Jeddah 22252, Saudi Arabia; dbokhary0001@stu.kau.edu.sa

**Keywords:** carbapenem-resistant enterobacteriaceae, microbiota, antibiotic resistance, ICU

## Abstract

Carbapenem-resistant Enterobacteriaceae (CRE) is a risk to public health worldwide and causes epidemic outbreaks in hospitals. The identification of alterations in the gut microbial profile can potentially serve as an early diagnostic tool to prevent harmful bacterial colonization. The purpose of this study was to characterize the gut microbiota profile of CRE-positive stool samples using 16S rRNA gene sequencing and to compare it with that of healthy control groups at King AbdulAziz University Hospital. Our results demonstrate that compared to the control group samples, the CRE-positive and CRE-negative group samples were less diverse and were dominated by a few operational taxonomic clusters of Enterococcus, Sphingomonas, and Staphylococcus. An analysis of samples from CRE-positive patients revealed Pseudomonas as the most abundant taxon. The existence of Pseudomonas in clinical samples undoubtedly indicates the development of resistance to a variety of antimicrobial drugs, with a less diverse microbiota. In our study, we found that the co-occurrence patterns of Klebsiella, Parabacteroides, Proteus and Pseudomonas differed between the CRE-negative and control stool groups.

## 1. Introduction

In 2019, the Centers for Disease Control and Prevention (CDC) classified 18 microorganisms according to the threat of antibiotic resistance into three categories: urgent, serious and concerning. Four bacteria are represented in the “urgent threats” category: Neisseria gonorrhoeae, Clostridium difficile, carbapenem-resistant Acinetobacter and carbapenem-resistant Enterobacter (CRE). The latter has been associated with more than 50% mortality among hospitalized patients [1,2]. In Saudi Arabia, increasing numbers of hospital CRE infection cases have been reported [3,4].

The predominant bacterial phyla in the healthy human gastrointestinal tract (GIT) are much more constant than in other body sites, dominated by Firmicutes and Bacteroidetes followed by Actinobacteria and Verrucomicrobia [5]. The abundance of Proteobacteria has been associated with inflammation [6,7]. However, the prevalence of uncultured gut species is still unknown [8].

Longitudinal data have shown that certain Enterobacteriaceae family members are more likely to reside in the human gut than others [9]; additionally, they have the ability to exchange antibiotic resistance and virulence genes [10]. Members of this family that establish fecal colonization include approximately 30 genera and 150 species of facultative anaerobes. Only 10 species are considered pathogens, such as E. coli, Citrobacter freundii, Proteus mirabilis and Klebsiella pneumoniae. Latency was identified in nosocomial outbreaks of strains harboring the NDM-1 carbapenemase [11]. Additionally, species such as Anaerotruncus colihominis in the family Ruminococcaceae are emerging, with serious clinical consequences [12].

In contrast, beneficial classes that could mediate resistance to infections are represented by the genera Lactobacillus, Bifidobacterium, Ruminococcus, Roseburia, Eubacterium, Barnesiella and Faecalibacterium [13,14]. These beneficial gut microbiota constituents can lower the occurrence of chronic inflammatory diseases, pathogenic infections and early death [15]. A disruption of the endogenous microbial community by antibiotics leads to the overgrowth of facultative anaerobes and increases epithelial permeability [16]. Five studies have concluded that antibiotic treatment tends to reduce the diversity of the microbiota, causing a dramatic reduction in the members of the gut microbiota in the Intensive Care Unit (ICU) environment [17,18,19,20,21].

The effect of broad-spectrum antibiotics has led to the overgrowth of Clostridioides difficile by reducing the capacity of the commensal microbiota to fight this pathogen [22]. For example, Bacteroidetes and Actinobacteria suppress exogenous C. difficile invasion [23]. Actinobacteria is reportedly more susceptible to carbapenem treatment than other phyla [24].

Previous studies have shown that the gut microbiota of the most extreme environment available for colonization are very complex. Our aim was to describe the gut microbiota of patients in the ICU and identify taxonomic markers associated with CRE carriers, as colonization precedes infection and the risk for developing infection among CRE carriers has yet to be determined.

## 2. Materials and Methods

### 2.1. Study Design and Sample Collection

The population was composed of 24 CRE-positive and 26 CRE-negative patients who were admitted to the Division of Internal Medicine (ICU) at King AbdulAziz University Hospital. The inclusion criteria for the CRE-positive and CRE-negative patients were applied upon hospitalization and every other week by routine screening for rectal CRE carriage by PCR for five genes only (KPC, NDM, OXA48, IMP, and VIM). Conventional identification was performed by blood and urine cultures for all patients, while stool, sputum and wound cultures were checked in some cases, and no pneumonia cases were detected between the two groups. Bacteremia was defined as positive blood growth only, as other results were different. The inclusion criteria for 10 healthy adults in the control group consisted of no antibiotic treatment for at least 6 months and no gastrointestinal disease prior to sampling. The clinical characteristics are presented in Table 1. Written informed consent was obtained from all participants. Fresh fecal samples were collected by the research team during ICU hospitalization. A total of 60 fecal samples were stored at −80 °C for subsequent DNA extraction.

This study was approved by the KAU Hospital Ethics Committee. Written informed consent was obtained from all participants.

### 2.2. Microbiota Sequencing and Taxonomy Assignment

Total DNA was extracted using (QIAGEN, Hilden, Germany) following the manufacturer’s protocol. Distinct regions (16S V4/16S V3/16S V3-V4) of the 16S rRNA/18S rRNA/ITS genes were amplified using amplicon generation and the specific primers 16SV34, 341F (CCTAYGGGRBGCASCAG), and 806R (GGACTACNNGGGTATCTAAT) with barcodes. All PCRs were carried out with Phusion^®^ [Editor2] High-Fidelity PCR Master Mix (New England Biolabs). The amplicon was sequenced on an Illumina paired-end platform to generate 250 bp paired-end raw reads (Raw PE), which were merged and pretreated to obtain clean tags. The chimeric sequences in the clean tags were detected and removed to obtain the effective tags that could be used for subsequent analysis. The FASTQ file was merged using the PEAR software package. Operational taxonomic units (OTUs) were defined based on 97% similarity clustering using the UCLUST algorithm. For each representative sequence, the Unite Database (https://unite.ut.ee/ (accessed on 7 February 2022) was used based on the Blast algorithm, which was calculated by QIIME software (Version 1.9.1) (http://qiime.org/scripts/assign_taxonomy.html (accessed on 25 March 2022)) to annotate taxonomic information.

The accepted paired-end, primer-trimmed reads were deposited at the National Center for Biotechnology Information (NCBI) under accession number SUB10994983.

### 2.3. Statistical Analysis

Within-sample (alpha) diversity was assessed using the observed taxa and Shannon index variation and tested with Wilcoxon (for two conditions) or Kruskal–Wallis and Dunn post hoc tests (for multiple conditions) [25,26]. Across-sample (beta) diversity measurements were performed to examine the sample dissimilarity by using the Aitchison distance, as described elsewhere [27], which is considered an appropriate method that takes into consideration the compositional nature of 16S rRNA sequencing data.

To assess the effect of each measured environmental factor on sample differences regarding microbial composition, PERMANOVA was used. Distance matrices were constructed based on an index of similarity of community membership and structure clustered based on Aitchison distance [27] to reveal major groups. All genera not filtered during gene copy number (GCN) correction were tested for differential abundance between any two sample types. Genera with significantly differential abundances were defined using a Venn diagram formalism with the following thresholds: |effect size| > 1 and adjusted *p*-value ≤ 0.05 for the expected Benjamini–Hochberg-corrected *p* value of Welch’s *t*-test (we.eBH) and/or expected Benjamini–Hochberg-corrected *p* value of Wilcoxon test (wi.eBH).

## 3. Results

### 3.1. Participants Profile

A total of 60 stool samples were assessed using 16S rRNA gene sequencing; only 10 stool samples were collected from the general population that was matched by age to the hospitalized groups. The rates of bacteremia between the two groups of hospitalized patients were similar, yet there was a higher rate of positive urine cultures in the CRE-positive group. A total of 27 participants in both the CRE-positive and CRE-negative groups died after stool samples were obtained. Antibiotic treatment was assessed based on broad- and narrow-spectrum antibiotics, and 23 patients in the CRE-positive group were on both types of antibiotics. The common broad-spectrum antibiotics used in this study included meropenem and piperacillin-tazobactam, while the narrow-spectrum antibiotics were colistin for multidrug-resistant (MDR) gram-negative sepsis and vancomycin for methicillin-resistant Staphylococcus aureus (MRSA) infections or severe suspected gram-positive sepsis.

### 3.2. Sequence Dataset Quality

The total DNA sequence was 1,051,849 bp, containing 2558 sequences ranging from 307 bp to 430 bp and averaging 411 bp in length. After the quality control pipeline was applied, 371 sequences were removed, and the total DNA sequence was 906,789 bp, containing 0 ambiguous bases and 2187 sequences ranging from 332 bp to 430 bp and averaging 414 bp in length (std. deviation from average length: 11.266). All these sequences had unique IDs. The average GC content was 53.474% (std. deviation: 3.210%), and the GC ratio was 0.877 (std. deviation: 0.114).

### 3.3. Microbial Diversity and Structure

**Figure 1:**
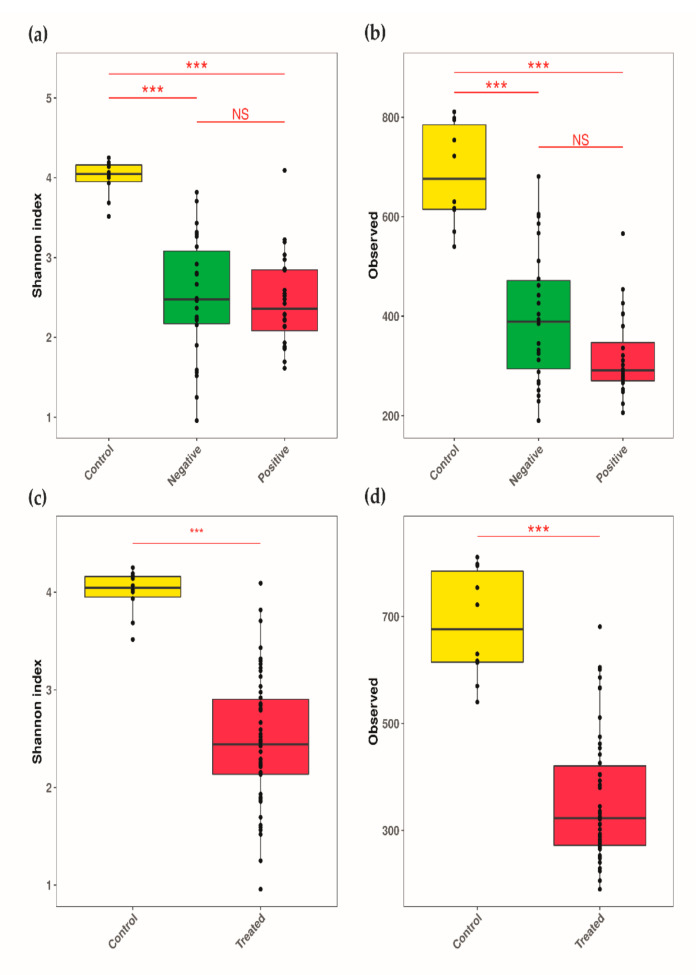
Boxplots based on observed species and Shannon indices showing the alpha-diversity of microbial communities. (**a**) Comparison of observed species between the CRE-based groups. (**b**) Comparison of Shannon indices between the CRE-based groups. (**c**) Comparison of observed species between antibiotic-treated and control samples. (**d**) Comparison of Shannon indices between antibiotic-treated and control samples. Within-sample diversity was compared between the CRE-based group samples using Kruskal–Wallis followed by Dunn’s post hoc test. Comparisons between antibiotic groups were performed using the Wilcoxon test. An adjusted or nominal *p*-value threshold of 0.05 was used to define statistical significance. *** (adjusted) *p*-value < 0.001; NS nonsignificant.

The variation in the bacterial diversity within the three groups, CRE-positive individuals, CRE-negative individuals and controls, was assessed by the Shannon index and number of observed taxa. The control group had a significantly higher bacterial richness than the other two groups (*p* < 0.005) (Figure 1a,b), and the same observation was true under the effect of antibiotic usage (Figure 1c,d).

**Figure 2:**
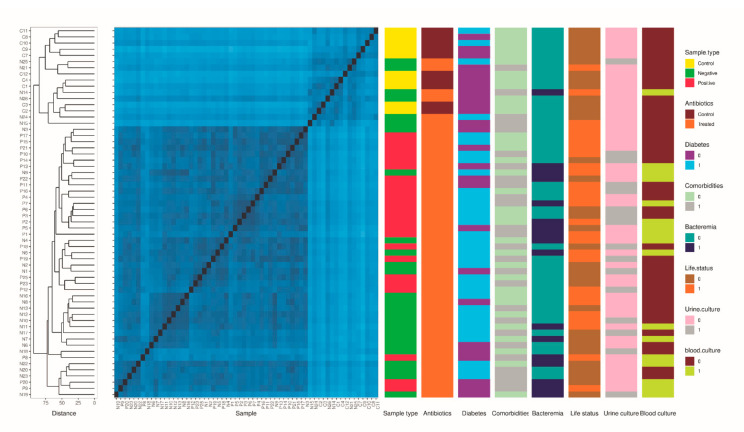
Beta diversity heatmap with hierarchical clustering of individual samples. Aitchison distance was used to hierarchically cluster individual samples using complete linkage.

The bacterial communities in the three groups were compared using the Atchison distance prior to constructing a heatmap. Compared with the CRE-based sample group and antibiotic-regimen-based groups, the control group tended to form a separate cluster along with a small number of CRE-negative group individuals treated with antibiotics (Figure 2).

The effect of confounding factors was assessed by PERMANOVA. CRE-positive and CRE-negative samples (R^2^: 0.240; *p*-value: 0.001) and antibiotics (R^2^: 0.186; *p*-value: 0.001) were the two factors most likely affecting individuals’ microbial compositions (Supplementary Table S1). Specifically, pairwise PERMANOVA revealed the greatest differences between CRE-positive samples and control samples (R^2^: 0.35; *p*-adjusted: 0.003), as well as between CRE-negative samples and control samples (R^2^: 0.20; *p*-adjusted: 0.003). Nevertheless, these observations can be at least partially explained by between-group heterogeneity of variance, except for CRE-negative sample versus control sample comparisons (Supplementary Figure S1a). The administered antibiotic formulations were also responsible for a proportion of the microbial variability observed (R^2^: 0.186; *p*-value: 0.001). A significant heterogeneity of variance was also found among the treated and untreated individuals, necessitating a careful interpretation of the findings (Supplementary Figure S1b).

### 3.4. Fecal Microbiome Taxonomy Differences between Samples Determined Using 16S rRNA Gene Sequencing

**Figure 3:**
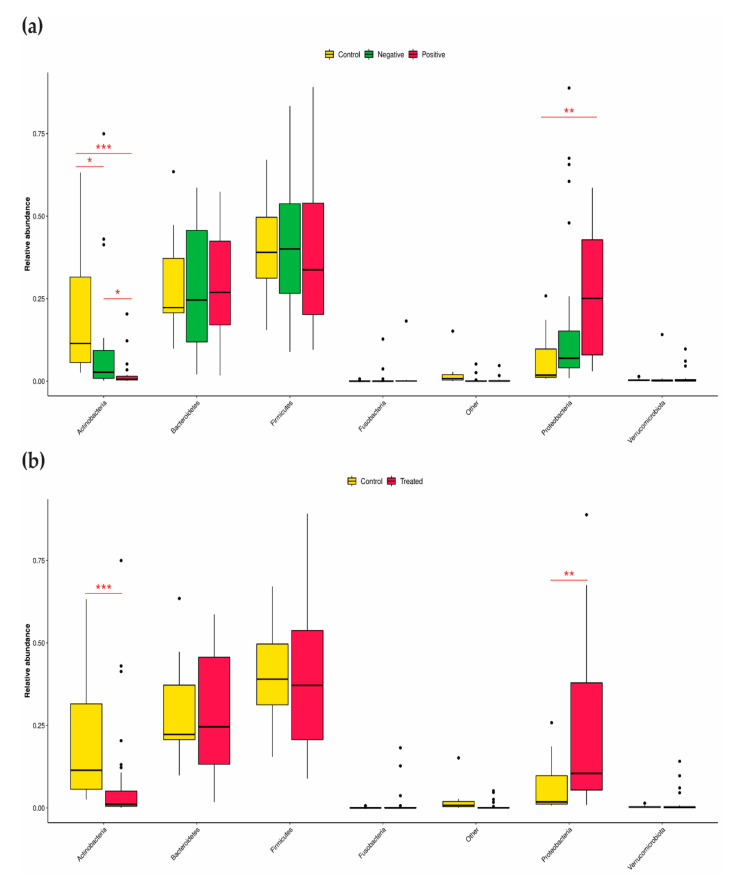
Relative abundance of phyla in the CRE-based and antibiotic regimen-based groups. (**a**) Relative abundance of the top phyla in CRE-based groups. (**b**) Relative abundance of the top phyla in antibiotic regimen-based groups. All taxa with a gene copy number-corrected relative abundance below the respective median were classified as “Other”. Differences in the relative abundance in the CRE-based groups were identified using the Kruskal–Wallis test followed by the Dunn post hoc test. Differences between the antibiotic groups were identified using the Wilcoxon test. An adjusted or nominal *p* value threshold of 0.05 was used to define statistical significance. * (adjusted) *p* value < 0.05; ** (adjusted) *p* value < 0.01; *** (adjusted) *p* value < 0.001.

The dominant bacterial phyla were *Bacteroidetes*, *Firmicutes* and *Proteobacteria* in all three groups (Supplementary Figure S2; Supplementary Table S2), and Actinobacteria represented the most abundant taxon in the control samples and the least abundant taxon in the CRE-negative samples. In contrast, in the CRE-positive group, Proteobacteria was the most abundant taxon (Figure 3a). Moreover, Actinobacteria and Proteobacteria were the only phyla significantly affected by antibiotic administration (Figure 3b). Both taxa had the same behavior between the conditions of both comparisons, suggesting a common response to steady state disruption.

**Figure 4:**
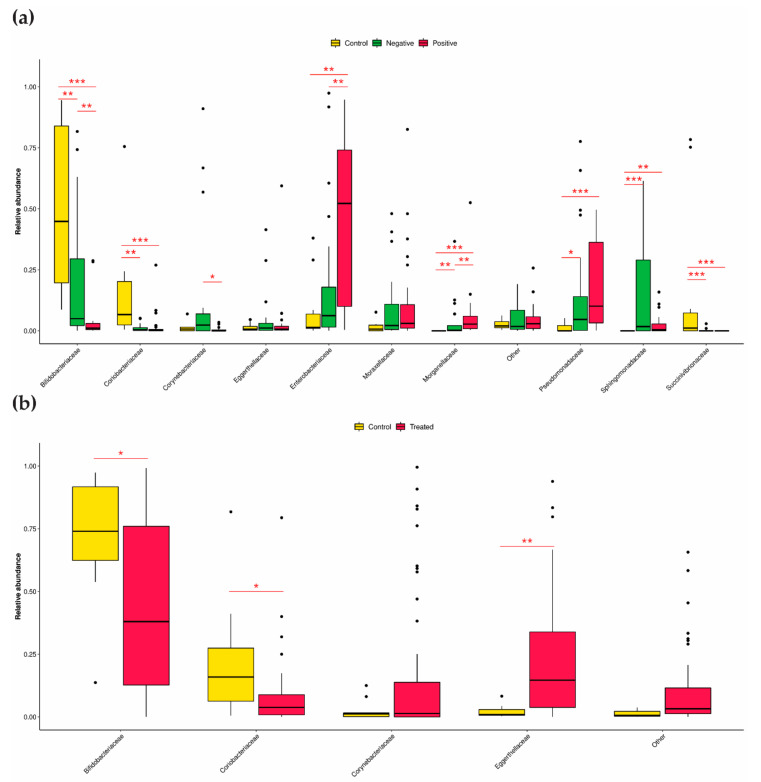
Relative abundance of families in the CRE-based and antibiotic regimen-based groups. (**a**) Relative abundance of the top families in the CRE-based groups. (**b**) Relative abundance of the top families in antibiotic regimen-based groups. All taxa with a gene copy number-corrected relative abundance below the respective 3rd quartile were classified as “Other”. Differences in the relative abundance of the CRE-based groups were identified using Kruskal–Wallis followed by Dunn’s post hoc test. Differences between the antibiotic groups were identified using the Wilcoxon test. An adjusted or nominal *p* value threshold of 0.05 was used to define statistical significance. * (adjusted) *p*-value < 0.05; ** (adjusted) *p*-value < 0.01; *** (adjusted) *p*-value < 0.001.

Further examination of families with increased abundance in the control samples (above the 3rd quartile) revealed the three taxa *Bifidobacteriaceae*, *Succinivibrionaceae* and *Coriobacteriaceae* (Figure 4a). The comparison of findings between the CRE-positive and CRE-negative groups revealed a significantly increased prevalence of the families *Enterobacteriaceae*, *Morganellaceae*, and *Pseudomonadaceae* and a significantly decreased prevalence of the families *Corynebacteriaceae* and *Sphingomonadaceae*. The abundance of the family *Corynebacteriaceae* seemed to be resistant to antibiotic regimens (Figure 4b).

**Figure 5:**
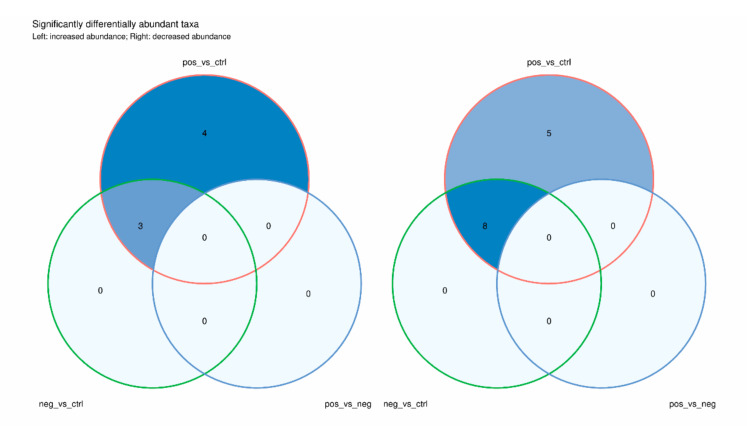
Common and unique genera among sample types. Venn diagram of differential abundance analysis performed between the CRE groups at the genus level. Genera found to be differentially abundant by at least one statistical test (Welch’s t or Wilcoxon test) are included. The analysis was performed on gene copy number-corrected values. An absolute ALDEx2 effect size > 1 and an adjusted *p*-value ≤ 0.05 were used as differential abundance thresholds.

At the genus level, we performed a differential abundance analysis using pairwise examination among the three groups, which revealed three taxa, *Enterococcus*, *Sphingomonas* and *Staphylococcus*, as the only genera with an increased abundance in both CRE-positive and CRE-negative samples compared to that in controls (Figure 5; Supplementary Table S3). The abundances of *Anaerostipes*, *Blautia*, *Collinsella*, *Dialister*, *Eubacterium*, *Faecalibacterium*, *Prevotella* and *Roseburia* changed in the opposite direction in both hospitalized groups compared to those in control groups.

Interestingly, in addition to the previous ones, four genera (*Klebsiella*, *Parabacteroides*, *Proteus*, and *Pseudomonas*) and five genera (*Bifidobacterium*, *Helicobacter*, *Lachnospira*, *Romboutsia*, and *Turicibacter*) were found to be enriched and depleted, respectively, in the CRE-positive stool samples compared to those in the nonhospitalized control group samples.

Finally, a sample comparison based on antibiotic administration revealed that drug treatments affected several genera in the same way (Supplementary Figure 3). Specifically, the abundance of *Enterococcus*, *Proteus*, *Sphingomonas* and *Staphylococcus* was increased during antibiotic treatment, while that of *Anaerostipes*, *Blautia*, *Collinsella*, *Dialister*, *Eubacterium*, *Faecalibacterium*, *Prevotella*, *Romboutsia* and *Roseburia* was decreased.

## 4. Discussion

New estimates from the World Health Organization (WHO) show a high mortality rate among patients with CRE infections due to both the severity of the infections and the lack of effective antibiotics, which requires intensive research for prevention [28]. The characterization of the gut microbial communities associated with antibiotic resistance, mostly MDR strains, would allow us to understand the deleterious effect on the host [29]. Broad-spectrum treatment diminishes the indigenous microbiota, thereby reducing resistance to colonization by pathogens [30]. The clinical consequence is the alteration of the immune defense against these harmful colonizers [14].

Our study is the second analysis of the gut microbiome in CRE-infected hospitalized patients under narrow- and broad-spectrum antibiotic management, complementing previous research by [31]. In that study, the enriched microbes found in the CRE carrier and noncarrier patients were *Klebsiella*, *Enterococcus*, and *Citrobacter*. In comparison, we distinguished similar and different microbial community structures among the three experimental groups. In the hospitalized groups, *Enterococcus*, *Sphingomonas* and *Staphylococcus* were the most dominant, and two taxa of *Citrobacter* were found to be relatively equally distributed in the CRE-positive group and enriched in the CRE-negative group. Additionally, reads related to members of the *Sphingomonadaceae* family were previously suggested to be considered part of the gut core microbiota and as active players in the maintenance of the immune response [32]; at this point, we cannot confirm whether the presence of this taxon mitigated other microbial invasion. In general, the genus *Sphingomonas* contains twelve known species, of which *Sphingomonas paucimobilis* [33] was previously reported in nosocomial infections, particularly among immunocompromised cases [34], and it was suggested to be a marker for hospital-contaminated environments [35]. Traditional microbiology misidentified the low-virulence aerobic gram-negative rod *Sphingomonas* in stool samples, and this should stimulate further investigation, for example, by using laser desorption ionization-time of flight mass spectrometry (MALDI-TOF MS) [36].

As expected, the richness of the microbiota in the CRE-negative group did not differ from that of the microbiota in the CRE-positive group since both groups received antibiotic treatments. Moreover, we did not observe clusters indicating different microbial structures for any of the experimental variables, as in the cases of diabetes mellitus. Previous studies have indicated a negative correlation between low butyrate-producing bacteria in the phylum *Firmicutes* and diabetes [37], and the same trend was observed under vancomycin treatment [38]. In contrast to our data from stool samples, the Firmicutes prevalence did not show abundance differences between the groups. Specifically, we found a high abundance of commensal bifidobacteria, which are butyrate-producing bacteria that have been shown to acquire antibiotic resistance genes for ecological survival [39].

The CRE-positive group showed low diversity (Figure 1) [40], with enrichments of *Klebsiella*, *Parabacteroides*, *Proteus* and *Pseudomonas,* compared to the other two groups. These taxa were suggested to be less susceptible to treatment [41].

*Pseudomonas* has been identified as a critical challenge for infection control in hospitals [42]. The co-occurrence of both opportunistic pathogens *Pseudomonas* and *Klebsiella* has been previously discussed [43]. Under selection pressure, these gram-negative bacteria tend to form biofilms at the site of infection and adjust their gene expression to use the carbon source and available iron for their benefit. Therefore, the restriction of carbon would disrupt biofilm formation by these pathogens.

One of the most controversial roles of the genus *Parabacteroides* in human health has been discussed elsewhere [44]; despite this genus being susceptible to carbapenems, emerging resistant clinical isolates have been reported [45].

Our controls had a higher microbial diversity, and their guts were enriched with protective flora (Figure 1a). In particular, the *Collinsella* genus was recently shown to have a negative correlation with severe cases of COVID-19 [46].

Previously, a study [47] revealed the different effects of antibiotic usage on ICU patients, as the administration of the broad-spectrum antibiotic piperacillin-tazobactam for severe nosocomial infections was shown to be harmful to the gut microbiota, and there were certain bacterial taxa that could be considered as markers for colonization, as this study found a depletion of the bacterial order *Clostridiales* along with the occurrence of carbapenem-resistant *Pseudomonas aeruginosa*. Additionally, some bacterial species coaccumulate, forming a complex network driven by metabolic production in a mutualistic symbiosis [48].

The outcome of gut dysbiosis under the effect of the treatments in this study led to a decrease in the diversity of several taxa, such as the indigenous *Turicibacter*, which induces the production of host serotonin [49]. Similar to the results in our study, combined treatments yielded a depletion of short-chain fatty acid (SCFA)-producing bacteria, *Succinivibrio* and *Prevotella*, and these SCFAs also act as anti-inflammatory agents [50]; in addition, an increase in the abundance of *Enterococcus* and *Staphylococcus* was observed. The coexistence of both genera in a particular ecological niche can be used to predict vancomycin-resistance gene transfer and potential emerging new isolates [51,52]. The other underreported commensal taxa of Corynebacterium in human gut microbiome studies remain a matter of debate regarding whether they are a contamination or imply clinical relevance [53].

Our data suggest that antibiotics have contributed to the disruption of the microbiota [54], causing gut dysbiosis of high/low relative abundance taxa of human gut commensal microbiota and pathogens. This study has some limitations, including the small number of participants in the control group, certain issues we faced regarding logistic manners, and stool quantity and quality. Additionally, value would have been added to the present study by combining the results of the carbapenem minimum inhibitory concentration (MIC) with the PCR results; we could not access these data because they are not provided on a routine basis by the laboratory. Further study is needed to evaluate larger groups of control samples and perhaps other possible factors, as the observational nature of our study allows the possibility of residual confounding.

## 5. Conclusions

Coevolution studies of the role played by microbial communities in regulating the human gut under selection pressure have been shown to be highly complex. Antibiotic management in the ICU has confirmed patterns of serious shifts toward low species richness and the overrepresentation of certain pathogens. Consistent with this, we clearly detected these pathogens in our stool analysis, and we observed other underestimated taxa that deserve further investigation in a metabolic context.

## Figures and Tables

**Table 1:** Clinical characteristics of the groups.

Variables	CRE-Positive Patients (n = 24)	CRE-Negative Patients (n = 26)	Controls (n = 10)
**Age**	61.8 ± 3.5 (23–89)	59.9 ± 3.4 (18–88)	58 ± 8.2 (20–90)
**Sex**			
Male	12	9	6
Female	12	17	4
**Combined treatments**			
Broad spectrum (meropenem and piperacillin-tazobactam)	22	23	
Narrow spectrum (vancomycin and colistin)	23	24	
**Comorbidities**			
GI disease			
Positive	6	3	
Negative	18	23	
Diabetes mellitus			
Positive	16	15	
Negative	8	11	
**Urine cultures**			
Positive	10	6	
Negative	14	20	
**Bacteremia (blood growth)**			0
Positive	9	8	
Negative	15	18	
**Length of stay in ICU**	29 ± 5.5	11.5 ± 2.5	0
**Outcome**			0
Death	15	12	
Discharge	7	11	

## Data Availability

The accepted paired-end, primer-trimmed reads were deposited at the National Center for Biotechnology Information (NCBI) under accession number SUB10994983.

## Supplementary Materials

The following supporting information can be downloaded at: https://www.mdpi.com/article/10.3390/microorganisms10071309/s1. Table S1. Detailed PERMANOVA statistics. Table S2. Phyla abundances. Table S3. Differential abundance analysis results. Figure S1. Between groups variance heterogeneity for PERMANOVA evaluation. Figure S2. Per sample top phyla relative abundance. Figure S3. Differentially abundant microbial genera between antibiotics.

## References

[1] van Loon K., Voor In ’t Holt A.F., Vos M.C. (2018). A Systematic Review and Meta-Analyses of the Clinical Epidemiology of Carbapenem-Resistant Enterobacteriaceae. Antimicrob. Agents Chemother..

[2] Chotiprasitsakul D., Srichatrapimuk S., Kirdlarp S., Pyden A.D., Santanirand P. (2019). Epidemiology of Carbapenem-Resistant Enterobacteriaceae: A 5-Year Experience at a Tertiary Care Hospital. Infect. Drug Resist..

[3] Alotaibi F. (2019). Carbapenem-Resistant Enterobacteriaceae: An Update Narrative Review from Saudi Arabia. J. Infect. Public Health.

[4] Garbati M.A., Sakkijha H., Abushaheen A. (2016). Infections Due to Carbapenem Resistant Enterobacteriaceae among Saudi Arabian Hospitalized Patients: A Matched Case-Control Study. BioMed Res. Int..

[5] Qin J., Li R., Raes J., Arumugam M., Burgdorf K.S., Manichanh C., Nielsen T., Pons N., Levenez F., Yamada T. (2010). A Human Gut Microbial Gene Catalogue Established by Metagenomic Sequencing. Nature.

[6] Frank D.N., St. Amand A.L., Feldman R.A., Boedeker E.C., Harpaz N., Pace N.R. (2007). Molecular-Phylogenetic Characterization of Microbial Community Imbalances in Human Inflammatory Bowel Diseases. Proc. Natl. Acad. Sci. USA.

[7] Shin N.-R., Whon T.W., Bae J.-W. (2015). Proteobacteria: Microbial Signature of Dysbiosis in Gut Microbiota. Trends Biotechnol..

[8] Almeida A., Mitchell A.L., Boland M., Forster S.C., Gloor G.B., Tarkowska A., Lawley T.D., Finn R.D. (2019). A New Genomic Blueprint of the Human Gut Microbiota. Nature.

[9] Martinson J.N.V., Pinkham N.V., Peters G.W., Cho H., Heng J., Rauch M., Broadaway S.C., Walk S.T. (2019). Rethinking Gut Microbiome Residency and the Enterobacteriaceae in Healthy Human Adults. ISME J..

[10] Goren M.G., Carmeli Y., Schwaber M.J., Chmelnitsky I., Schechner V., Navon-Venezia S. (2010). Transfer of Carbapenem-Resistant Plasmid from Klebsiella Pneumoniae ST258 to Escherichia Coli in Patient. Emerg. Infect. Dis..

[11] Voulgari E., Gartzonika C., Vrioni G., Politi L., Priavali E., Levidiotou-Stefanou S., Tsakris A. (2014). The Balkan Region: NDM-1-Producing Klebsiella Pneumoniae ST11 Clonal Strain Causing Outbreaks in Greece. J. Antimicrob. Chemother..

[12] Lau S.K.P., Woo P.C.Y., Woo G.K.S., Fung A.M.Y., Ngan A.H.Y., Song Y., Liu C., Summanen P., Finegold S.M., Yuen K. (2006). Bacteraemia Caused by Anaerotruncus Colihominis and Emended Description of the Species. J. Clin. Pathol..

[13] Fukuda S., Toh H., Hase K., Oshima K., Nakanishi Y., Yoshimura K., Tobe T., Clarke J.M., Topping D.L., Suzuki T. (2011). Bifidobacteria Can Protect from Enteropathogenic Infection through Production of Acetate. Nature.

[14] Ubeda C., Pamer E.G. (2012). Antibiotics, Microbiota, and Immune Defense. Trends Immunol..

[15] Rampelli S., Soverini M., D’Amico F., Barone M., Tavella T., Monti D., Capri M., Astolfi A., Brigidi P., Biagi E. (2020). Shotgun Metagenomics of Gut Microbiota in Humans with up to Extreme Longevity and the Increasing Role of Xenobiotic Degradation. Msystems.

[16] Zoetendal E.G., Collier C.T., Koike S., Mackie R.I., Gaskins H.R. (2004). Molecular Ecological Analysis of the Gastrointestinal Microbiota: A Review. J. Nutr..

[17] Buelow E., Bello González T.d.j., Fuentes S., de Steenhuijsen Piters W.A.A., Lahti L., Bayjanov J.R., Majoor E.A.M., Braat J.C., van Mourik M.S.M., Oostdijk E.A.N. (2017). Comparative Gut Microbiota and Resistome Profiling of Intensive Care Patients Receiving Selective Digestive Tract Decontamination and Healthy Subjects. Microbiome.

[18] Yeh A., Rogers M.B., Firek B., Neal M.D., Zuckerbraun B.S., Morowitz M.J. (2016). Dysbiosis Across Multiple Body Sites in Critically Ill Adult Surgical Patients. Shock. Inj. Inflamm. Sepsis Lab. Clin. Approaches.

[19] McDonald D., Ackermann G., Khailova L., Baird C., Heyland D., Kozar R., Lemieux M., Derenski K., King J., Vis-Kampen C. (2016). Extreme Dysbiosis of the Microbiome in Critical Illness. Msphere.

[20] Ojima M., Motooka D., Shimizu K., Gotoh K., Shintani A., Yoshiya K., Nakamura S., Ogura H., Iida T., Shimazu T. (2016). Metagenomic Analysis Reveals Dynamic Changes of Whole Gut Microbiota in the Acute Phase of Intensive Care Unit Patients. Dig. Dis. Sci..

[21] Zaborin A., Smith D., Garfield K., Quensen J., Shakhsheer B., Kade M., Tirrell M., Tiedje J., Gilbert J.A., Zaborina O. (2014). Membership and Behavior of Ultra-Low-Diversity Pathogen Communities Present in the Gut of Humans during Prolonged Critical Illness. mBio.

[22] Jernberg C., Löfmark S., Edlund C., Jansson J.K. (2010). Long-Term Impacts of Antibiotic Exposure on the Human Intestinal Microbiota. Microbiol. Read. Engl..

[23] Moya A., Ferrer M. (2016). Functional Redundancy-Induced Stability of Gut Microbiota Subjected to Disturbance. Trends Microbiol..

[24] Ojima M., Shimizu K., Motooka D., Ishihara T., Nakamura S., Shintani A., Ogura H., Iida T., Yoshiya K., Shimazu T. (2022). Gut Dysbiosis Associated with Antibiotics and Disease Severity and Its Relation to Mortality in Critically Ill Patients. Dig. Dis. Sci..

[25] Costello E.K., Lauber C.L., Hamady M., Fierer N., Gordon J.I., Knight R. (2009). Bacterial Community Variation in Human Body Habitats across Space and Time. Science.

[26] Turnbaugh P.J., Quince C., Faith J.J., McHardy A.C., Yatsunenko T., Niazi F., Affourtit J., Egholm M., Henrissat B., Knight R. (2010). Organismal, Genetic, and Transcriptional Variation in the Deeply Sequenced Gut Microbiomes of Identical Twins. Proc. Natl. Acad. Sci. USA.

[27] Gloor G.B., Macklaim J.M., Pawlowsky-Glahn V., Egozcue J.J. (2017). Microbiome Datasets Are Compositional: And This Is Not Optional. Front. Microbiol..

[28] Tomczyk S., Zanichelli V., Grayson M.L., Twyman A., Abbas M., Pires D., Allegranzi B., Harbarth S. (2019). Control of Carbapenem-Resistant Enterobacteriaceae, Acinetobacter Baumannii, and Pseudomonas Aeruginosa in Healthcare Facilities: A Systematic Review and Reanalysis of Quasi-Experimental Studies. Clin. Infect. Dis. Off. Publ. Infect. Dis. Soc. Am..

[29] Arias C.A., Murray B.E. (2012). The Rise of the Enterococcus: Beyond Vancomycin Resistance. Nat. Rev. Microbiol..

[30] Panda S., El khader I., Casellas F., López Vivancos J., García Cors M., Santiago A., Cuenca S., Guarner F., Manichanh C. (2014). Short-Term Effect of Antibiotics on Human Gut Microbiota. PLoS ONE.

[31] Korach-Rechtman H., Hreish M., Fried C., Gerassy-Vainberg S., Azzam Z.S., Kashi Y., Berger G. (2020). Intestinal Dysbiosis in Carriers of Carbapenem-Resistant Enterobacteriaceae. Msphere.

[32] D’Auria G., Peris-Bondia F., Džunková M., Mira A., Collado M.C., Latorre A., Moya A. (2013). Active and Secreted IgA-Coated Bacterial Fractions from the Human Gut Reveal an under-Represented Microbiota Core. Sci. Rep..

[33] Kuo I.-C., Lu P.-L., Lin W.-R., Lin C.-Y., Chang Y.-W., Chen T.-C., Chen Y.-H. (2009). Sphingomonas Paucimobilis Bacteraemia and Septic Arthritis in a Diabetic Patient Presenting with Septic Pulmonary Emboli. J. Med. Microbiol..

[34] Pascale R., Russo E., Esposito I., Leone S., Esposito S. (2013). Sphingomonas Paucimobilis Osteomyelitis in an Immunocompetent Patient. A Rare Case Report and Literature Review. New Microbiol..

[35] Holmes B., Owen R.J., Evans A., Malnick H., Willcox W.R.Y. (1977). Pseudomonas Paucimobilis, a New Species Isolated from Human Clinical Specimens, the Hospital Environment, and Other Sources. Int. J. Syst. Evol. Microbiol..

[36] Halden R.U., Colquhoun D.R., Wisniewski E.S. (2005). Identification and Phenotypic Characterization of Sphingomonas Wittichii Strain RW1 by Peptide Mass Fingerprinting Using Matrix-Assisted Laser Desorption Ionization-Time of Flight Mass Spectrometry. Appl. Environ. Microbiol..

[37] Tang W.H.W., Kitai T., Hazen S.L. (2017). Gut Microbiota in Cardiovascular Health and Disease. Circ. Res..

[38] Vrieze A., Out C., Fuentes S., Jonker L., Reuling I., Kootte R.S., van Nood E., Holleman F., Knaapen M., Romijn J.A. (2014). Impact of Oral Vancomycin on Gut Microbiota, Bile Acid Metabolism, and Insulin Sensitivity. J. Hepatol..

[39] Duranti S., Lugli G.A., Mancabelli L., Turroni F., Milani C., Mangifesta M., Ferrario C., Anzalone R., Viappiani A., van Sinderen D. (2017). Prevalence of Antibiotic Resistance Genes among Human Gut-Derived Bifidobacteria. Appl. Environ. Microbiol..

[40] Sommer F., Anderson J.M., Bharti R., Raes J., Rosenstiel P. (2017). The Resilience of the Intestinal Microbiota Influences Health and Disease. Nat. Rev. Microbiol..

[41] Pérez-Cobas A.E., Artacho A., Knecht H., Ferrús M.L., Friedrichs A., Ott S.J., Moya A., Latorre A., Gosalbes M.J. (2013). Differential Effects of Antibiotic Therapy on the Structure and Function of Human Gut Microbiota. PLoS ONE.

[42] Harris A.D., Jackson S.S., Robinson G., Pineles L., Leekha S., Thom K.A., Wang Y., Doll M., Pettigrew M.M., Johnson J.K. (2016). Pseudomonas Aeruginosa Colonization in the Intensive Care Unit: Prevalence, Risk Factors, and Clinical Outcomes. Infect. Control Hosp. Epidemiol..

[43] Riquelme S.A., Ahn D., Prince A. (2018). Pseudomonas Aeruginosa and Klebsiella Pneumoniae Adaptation to Innate Immune Clearance Mechanisms in the Lung. J. Innate Immun..

[44] Ezeji J.C., Sarikonda D.K., Hopperton A., Erkkila H.L., Cohen D.E., Martinez S.P., Cominelli F., Kuwahara T., Dichosa A.E.K., Good C.E. (2021). Parabacteroides Distasonis: Intriguing Aerotolerant Gut Anaerobe with Emerging Antimicrobial Resistance and Pathogenic and Probiotic Roles in Human Health. Gut Microbes.

[45] Byun J.H., Kim M., Lee Y., Lee K., Chong Y. (2019). Antimicrobial Susceptibility Patterns of Anaerobic Bacterial Clinical Isolates From 2014 to 2016, Including Recently Named or Renamed Species. Ann. Lab. Med..

[46] Hirayama M., Nishiwaki H., Hamaguchi T., Ito M., Ueyama J., Maeda T., Kashihara K., Tsuboi Y., Ohno K. (2021). Intestinal Collinsella May Mitigate Infection and Exacerbation of COVID-19 by Producing Ursodeoxycholate. PLoS ONE.

[47] Pettigrew M.M., Gent J.F., Kong Y., Halpin A.L., Pineles L., Harris A.D., Johnson J.K. (2019). Gastrointestinal Microbiota Disruption and Risk of Colonization With Carbapenem-Resistant Pseudomonas Aeruginosa in Intensive Care Unit Patients. Clin. Infect. Dis..

[48] Willing B.P., Russell S.L., Finlay B.B. (2011). Shifting the Balance: Antibiotic Effects on Host–Microbiota Mutualism. Nat. Rev. Microbiol..

[49] Fung T.C., Vuong H.E., Luna C.D.G., Pronovost G.N., Aleksandrova A.A., Riley N.G., Vavilina A., McGinn J., Rendon T., Forrest L.R. (2019). Intestinal Serotonin and Fluoxetine Exposure Modulate Bacterial Colonization in the Gut. Nat. Microbiol..

[50] den Besten G., van Eunen K., Groen A.K., Venema K., Reijngoud D.-J., Bakker B.M. (2013). The Role of Short-Chain Fatty Acids in the Interplay between Diet, Gut Microbiota, and Host Energy Metabolism. J. Lipid Res..

[51] Ray A.J., Pultz N.J., Bhalla A., Aron D.C., Donskey C.J. (2003). Coexistence of Vancomycin-Resistant Enterococci and Staphylococcus Aureus in the Intestinal Tracts of Hospitalized Patients. Clin. Infect. Dis..

[52] Squier C., Rihs J.D., Risa K.J., Sagnimeni A., Wagener M.M., Stout J., Muder R.R., Singh N. (2002). Staphylococcus Aureus Rectal Carriage and Its Association with Infections in Patients in a Surgical Intensive Care Unit and a Liver Transplant Unit. Infect. Control Hosp. Epidemiol..

[53] Bernard K. (2012). The Genus Corynebacterium and Other Medically Relevant Coryneform-like Bacteria. J. Clin. Microbiol..

[54] Ramirez J., Guarner F., Fernandez L.B., Maruy A., Sdepanian V.L., Cohen H. (2020). Antibiotics as Major Disruptors of Gut Microbiota. Front. Cell. Infect. Microbiol..

