# Supplementary material for: Profile of the Gut Microbiome Containing Carbapenem-Resistant Enterobacteriaceae in ICU Patients
Source: Microorganisms. 2022 Jun 28;10(7):1309. doi: 10.3390/microorganisms10071309 (PMC9320093; doi:10.3390/microorganisms10071309)
Supplement: Supplementary file 1 [file microorganisms-10-01309-s001.zip › microorganisms-1747238-supplementary.pdf]

Profile of the Gut Microbiome Containing Carbapenem-Resistant *Enterobacteriaceae* in ICU patients.

Anees A. Sindi, Sarah M. Alsayed, Ibrahim Abushoshah , Diyaa H. Bokhary , Nisreen R Tashkandy.

Department of Anesthesia and Critical Care, Faculty of Medicine, King AbdulAziz University Jeddah, Saudi Arabia asindi2@kau.edu.sa

Department of Biological Sciences Faculty of science, King AbdulAziz University Jeddah, Saudi Arabia. sabdulazizalsayed@stu.kau.edu.sa

Department of Anesthesia and Critical Care Faculty of Medicine King Abdulaziz University Jeddah, Saudi Arabia. Iabushoshah@kau.edu.sa

Department of Emergency Medicine, King Abdulaziz University Hospital Jeddah, Saudi Arabia. dbokhary0001@stu.kau.edu.sa

Department of Biological Sciences Faculty of science, King Abdulaziz University Jeddah, Saudi Arabia. ntashkandy@kau.edu.sa

Supplementary Data

Supplementary Tables

Supplementary Table S1: Detailed PERMANOVA statistics

Variable	R^2	value	Variance. Heterogeneity
Sample. Type	0.240233281	0.001	Yes
Antibiotics	0.213700951	0.001	No
Bacteremia	0.035634014	0.034	Yes
Diabetes.mellitus	0.065964659	0.001	Yes
Comorbidities	0.030434444	0.047	No
Life. Status	0.06160379	0.004	Yes
Urine. Culture	0.025367912	0.102	No
Blood. Culture	0.035634014	0.034	Yes

Supplementary Table S2: Phyla abundances

Sample	Phylum	Relative Abundance
P9	Bacteroidetes	0.57429078
P9	Firmicutes	0.353634752
P9	Proteobacteria	0.059219858
P9	Verrucomicrobiota	0.008865248
P9	Other	0.002039007
P9	Actinobacteria	0.00106383
P9	Fusobacteria	0.000886525
P8	Bacteroidetes	0.557401531
P8	Firmicutes	0.282714206
P8	Proteobacteria	0.099309315
P8	Verrucomicrobiota	0.04601456
P8	Actinobacteria	0.009893597
P8	Fusobacteria	0.002613403
P8	Other	0.002053388
P7	Proteobacteria	0.511212091
P7	Firmicutes	0.217820804
P7	Actinobacteria	0.203694557
P7	Bacteroidetes	0.061641806
P7	Fusobacteria	0.003655043
P7	Verrucomicrobiota	0.001679344
P7	Other	0.000296355

P6	Firmicutes	0.890996785
P6	Proteobacteria	0.086173633
P6	Bacteroidetes	0.017577706
P6	Actinobacteria	0.003644159
P6	Verrucomicrobiota	0.000857449
P6	Other	0.000750268
P6	Fusobacteria	0
P5	Bacteroidetes	0.472386776
P5	Proteobacteria	0.379801261
P5	Firmicutes	0.142365756
P5	Actinobacteria	0.004108542
P5	Verrucomicrobiota	0.00076438
P5	Other	0.000573285
P5	Fusobacteria	0
P4	Proteobacteria	0.426669577
P4	Bacteroidetes	0.39513313
P4	Firmicutes	0.121235268
P4	Other	0.047250109
P4	Actinobacteria	0.006765605
P4	Verrucomicrobiota	0.002837189
P4	Fusobacteria	0.000109123
P3	Firmicutes	0.508563187
P3	Bacteroidetes	0.401297999
P3	Proteobacteria	0.054083288
P3	Actinobacteria	0.034342888
P3	Verrucomicrobiota	0.001261943
P3	Other	0.000450694
P3	Fusobacteria	0
P25	Firmicutes	0.633109196
P25	Bacteroidetes	0.301070177
P25	Proteobacteria	0.060138271
P25	Actinobacteria	0.004451179
P25	Verrucomicrobiota	0.001231177
P25	Fusobacteria	0
P25	Other	0
P23	Firmicutes	0.729972646

P23	Bacteroidetes	0.220691676
P23	Proteobacteria	0.038686987
P23	Actinobacteria	0.010257913
P23	Verrucomicrobiota	0.000293083
P23	Fusobacteria	9.77E-05
P23	Other	0
P22	Bacteroidetes	0.56329588
P22	Firmicutes	0.321629213
P22	Proteobacteria	0.112078652
P22	Actinobacteria	0.002434457
P22	Other	0.000561798
P22	Fusobacteria	0
P22	Verrucomicrobiota	0
P21	Proteobacteria	0.492402706
P21	Firmicutes	0.455935693
P21	Bacteroidetes	0.033134006
P21	Actinobacteria	0.018527595
P21	Fusobacteria	0
P21	Other	0
P21	Verrucomicrobiota	0
P20	Firmicutes	0.784899511
P20	Bacteroidetes	0.171917436
P20	Proteobacteria	0.030599312
P20	Actinobacteria	0.005069708
P20	Verrucomicrobiota	0.004617056
P20	Other	0.002896976
P20	Fusobacteria	0
P2	Firmicutes	0.453615128
P2	Proteobacteria	0.299888765
P2	Bacteroidetes	0.237041157
P2	Verrucomicrobiota	0.00567297
P2	Actinobacteria	0.003448276
P2	Other	0.000333704
P2	Fusobacteria	0
P19	Bacteroidetes	0.348060459
P19	Proteobacteria	0.269633231

P19	Firmicutes	0.195856675
P19	Fusobacteria	0.18232745
P19	Actinobacteria	0.003699398
P19	Verrucomicrobiota	0.000317091
P19	Other	0.000105697
P18	Proteobacteria	0.561228335
P18	Bacteroidetes	0.215712133
P18	Firmicutes	0.206574981
P18	Actinobacteria	0.014506405
P18	Verrucomicrobiota	0.001130369
P18	Fusobacteria	0.00075358
P18	Other	9.42E-05
P17	Firmicutes	0.480940715
P17	Proteobacteria	0.448799608
P17	Bacteroidetes	0.059578638
P17	Other	0.004409603
P17	Actinobacteria	0.002645762
P17	Fusobacteria	0.002547771
P17	Verrucomicrobiota	0.001077903
P16	Proteobacteria	0.364710813
P16	Bacteroidetes	0.335456832
P16	Firmicutes	0.289019279
P16	Actinobacteria	0.004610226
P16	Other	0.002430847
P16	Verrucomicrobiota	0.00217938
P16	Fusobacteria	0.001592624
P15	Proteobacteria	0.585724283
P15	Bacteroidetes	0.235129461
P15	Firmicutes	0.162651205
P15	Actinobacteria	0.011696491
P15	Fusobacteria	0.00369889
P15	Verrucomicrobiota	0.0009997
P15	Other	1.00E-04
P14	Proteobacteria	0.434120208
P14	Bacteroidetes	0.408421229
P14	Firmicutes	0.095010882

P14	Verrucomicrobiota	0.060857191
P14	Actinobacteria	0.00100452
P14	Fusobacteria	0.00058597
P14	Other	0
P13	Bacteroidetes	0.506754024
P13	Proteobacteria	0.197524873
P13	Firmicutes	0.18272264
P13	Verrucomicrobiota	0.097630025
P13	Actinobacteria	0.013427162
P13	Fusobacteria	0.001779503
P13	Other	0.000161773
P12	Firmicutes	0.707552637
P12	Bacteroidetes	0.132316013
P12	Proteobacteria	0.101506664
P12	Actinobacteria	0.052057176
P12	Verrucomicrobiota	0.003959822
P12	Fusobacteria	0.00183504
P12	Other	0.000772648
P11	Bacteroidetes	0.538570849
P11	Proteobacteria	0.231729598
P11	Firmicutes	0.203816484
P11	Other	0.017153877
P11	Actinobacteria	0.005278116
P11	Verrucomicrobiota	0.002233049
P11	Fusobacteria	0.001218027
P10	Firmicutes	0.406170966
P10	Proteobacteria	0.37814871
P10	Bacteroidetes	0.196965099
P10	Actinobacteria	0.016489631
P10	Fusobacteria	0.001416287
P10	Verrucomicrobiota	0.000708144
P10	Other	0.000101163
P1	Firmicutes	0.661587237
P1	Bacteroidetes	0.166803027
P1	Actinobacteria	0.122213132
P1	Proteobacteria	0.041828595

P1	Verrucomicrobiota	0.006136224
P1	Other	0.001022704
P1	Fusobacteria	0.000409082
N9	Firmicutes	0.820466321
N9	Bacteroidetes	0.098272884
N9	Proteobacteria	0.054835924
N9	Actinobacteria	0.025647668
N9	Other	0.000690846
N9	Verrucomicrobiota	8.64E-05
N9	Fusobacteria	0
N8	Actinobacteria	0.749656677
N8	Firmicutes	0.156959367
N8	Proteobacteria	0.070037968
N8	Bacteroidetes	0.023265207
N8	Verrucomicrobiota	8.08E-05
N8	Fusobacteria	0
N8	Other	0
N7	Bacteroidetes	0.576510892
N7	Firmicutes	0.298049895
N7	Proteobacteria	0.112262825
N7	Actinobacteria	0.008608573
N7	Verrucomicrobiota	0.003338018
N7	Other	0.001229796
N7	Fusobacteria	0
N6	Bacteroidetes	0.586276659
N6	Firmicutes	0.364073169
N6	Proteobacteria	0.036584338
N6	Actinobacteria	0.008429571
N6	Verrucomicrobiota	0.004551968
N6	Other	8.43E-05
N6	Fusobacteria	0
N5	Firmicutes	0.506291764
N5	Bacteroidetes	0.460557147
N5	Proteobacteria	0.025080274
N5	Actinobacteria	0.005814458
N5	Fusobacteria	0.002256357

N5	Other	0
N5	Verrucomicrobiota	0
N4	Firmicutes	0.541991125
N4	Bacteroidetes	0.444611907
N4	Proteobacteria	0.009294147
N4	Actinobacteria	0.004102822
N4	Fusobacteria	0
N4	Other	0
N4	Verrucomicrobiota	0
N3	Proteobacteria	0.656509963
N3	Firmicutes	0.208164055
N3	Bacteroidetes	0.118204682
N3	Actinobacteria	0.01325208
N3	Fusobacteria	0.00386922
N3	Other	0
N3	Verrucomicrobiota	0
N26	Proteobacteria	0.479604881
N26	Firmicutes	0.261359675
N26	Bacteroidetes	0.245322487
N26	Actinobacteria	0.011272516
N26	Other	0.001394538
N26	Fusobacteria	0.000813481
N26	Verrucomicrobiota	0.000232423
N25	Proteobacteria	0.675473268
N25	Firmicutes	0.153214063
N25	Bacteroidetes	0.11972124
N25	Actinobacteria	0.038693572
N25	Fusobacteria	0.006864989
N25	Verrucomicrobiota	0.003328479
N25	Other	0.002704389
N24	Bacteroidetes	0.509908945
N24	Firmicutes	0.353686842
N24	Actinobacteria	0.107570077
N24	Proteobacteria	0.025620425
N24	Verrucomicrobiota	0.002142475
N24	Other	0.001071237

N24	Fusobacteria	0
N23	Firmicutes	0.509679204
N23	Bacteroidetes	0.357485251
N23	Actinobacteria	0.047658555
N23	Proteobacteria	0.044893068
N23	Fusobacteria	0.037334071
N23	Verrucomicrobiota	0.001567109
N23	Other	0.001382743
N22	Proteobacteria	0.888238806
N22	Firmicutes	0.088835821
N22	Bacteroidetes	0.020179104
N22	Actinobacteria	0.001910448
N22	Verrucomicrobiota	0.000358209
N22	Fusobacteria	0.000238806
N22	Other	0.000238806
N21	Bacteroidetes	0.543485054
N21	Firmicutes	0.204457869
N21	Fusobacteria	0.12748672
N21	Actinobacteria	0.104468285
N21	Proteobacteria	0.016769087
N21	Other	0.001978961
N21	Verrucomicrobiota	0.001354026
N20	Firmicutes	0.523841504
N20	Bacteroidetes	0.235440852
N20	Proteobacteria	0.15648086
N20	Actinobacteria	0.082605776
N20	Other	0.000767533
N20	Fusobacteria	0.000479708
N20	Verrucomicrobiota	0.000383767
N2	Firmicutes	0.422781091
N2	Bacteroidetes	0.345346124
N2	Proteobacteria	0.138708841
N2	Actinobacteria	0.090830525
N2	Other	0.00146919
N2	Verrucomicrobiota	0.000777807
N2	Fusobacteria	8.64E-05

N19	Firmicutes	0.582197543
N19	Bacteroidetes	0.368837288
N19	Other	0.025912839
N19	Proteobacteria	0.010432442
N19	Actinobacteria	0.008244994
N19	Verrucomicrobiota	0.003870099
N19	Fusobacteria	0.000504796
N18	Firmicutes	0.488433515
N18	Bacteroidetes	0.314116576
N18	Verrucomicrobiota	0.141347905
N18	Proteobacteria	0.04845173
N18	Actinobacteria	0.006284153
N18	Other	0.001275046
N18	Fusobacteria	9.11E-05
N17	Bacteroidetes	0.499949151
N17	Firmicutes	0.378826401
N17	Actinobacteria	0.059290145
N17	Proteobacteria	0.05898505
N17	Fusobacteria	0.002949253
N17	Other	0
N17	Verrucomicrobiota	0
N16	Firmicutes	0.73339681
N16	Actinobacteria	0.131159248
N16	Bacteroidetes	0.071887646
N16	Proteobacteria	0.055224946
N16	Verrucomicrobiota	0.00833135
N16	Fusobacteria	0
N16	Other	0
N15	Actinobacteria	0.413598048
N15	Firmicutes	0.290039929
N15	Bacteroidetes	0.132542147
N15	Proteobacteria	0.107586513
N15	Other	0.052018634
N15	Verrucomicrobiota	0.004214729
N15	Fusobacteria	0
N14	Actinobacteria	0.430310408

N14	Firmicutes	0.281019042
N14	Proteobacteria	0.257108078
N14	Bacteroidetes	0.023824015
N14	Verrucomicrobiota	0.003912703
N14	Other	0.003825754
N14	Fusobacteria	0
N13	Firmicutes	0.833706318
N13	Proteobacteria	0.076605268
N13	Bacteroidetes	0.069719401
N13	Actinobacteria	0.015321054
N13	Verrucomicrobiota	0.00464796
N13	Fusobacteria	0
N13	Other	0
N12	Bacteroidetes	0.477667948
N12	Firmicutes	0.438952956
N12	Proteobacteria	0.069009702
N12	Actinobacteria	0.010342303
N12	Verrucomicrobiota	0.004027091
N12	Fusobacteria	0
N12	Other	0
N11	Firmicutes	0.57529951
N11	Bacteroidetes	0.246522473
N11	Actinobacteria	0.093752513
N11	Proteobacteria	0.082415373
N11	Verrucomicrobiota	0.001608105
N11	Fusobacteria	0.000402026
N11	Other	0
N10	Firmicutes	0.709696609
N10	Bacteroidetes	0.242967621
N10	Proteobacteria	0.03892241
N10	Actinobacteria	0.007648509
N10	Verrucomicrobiota	0.000764851
N10	Fusobacteria	0
N10	Other	0
N1	Proteobacteria	0.605400458
N1	Bacteroidetes	0.189656751

N1	Firmicutes	0.175743707
N1	Actinobacteria	0.028741419
N1	Fusobacteria	0.0002746
N1	Verrucomicrobiota	0.000183066
N1	Other	0
C9	Firmicutes	0.511100558
C9	Bacteroidetes	0.228303455
C9	Proteobacteria	0.186156951
C9	Actinobacteria	0.046539238
C9	Other	0.01911433
C9	Verrucomicrobiota	0.008548023
C9	Fusobacteria	0.000237445
C8	Firmicutes	0.375017121
C8	Bacteroidetes	0.205451308
C8	Actinobacteria	0.156279962
C8	Other	0.151623065
C8	Proteobacteria	0.107108615
C8	Verrucomicrobiota	0.004519929
C8	Fusobacteria	0
C7	Bacteroidetes	0.634590734
C7	Firmicutes	0.215802966
C7	Actinobacteria	0.13211866
C7	Proteobacteria	0.01034609
C7	Other	0.003479216
C7	Verrucomicrobiota	0.0032961
C7	Fusobacteria	0.000366233
C4	Firmicutes	0.40558524
C4	Actinobacteria	0.36838391
C4	Bacteroidetes	0.21231979
C4	Proteobacteria	0.008367779
C4	Verrucomicrobiota	0.002520415
C4	Fusobacteria	0.001814699
C4	Other	0.001008166
C3	Firmicutes	0.67109744
C3	Bacteroidetes	0.217400857
C3	Actinobacteria	0.086674723

C3	Verrucomicrobiota	0.01439086
C3	Proteobacteria	0.007140503
C3	Other	0.003295617
C3	Fusobacteria	0
C2	Firmicutes	0.557266153
C2	Bacteroidetes	0.324935853
C2	Actinobacteria	0.096337765
C2	Proteobacteria	0.016561698
C2	Other	0.003732214
C2	Verrucomicrobiota	0.000816422
C2	Fusobacteria	0.000349895
C12	Bacteroidetes	0.387982276
C12	Firmicutes	0.296444396
C12	Proteobacteria	0.258294607
C12	Actinobacteria	0.036312547
C12	Other	0.011996109
C12	Fusobacteria	0.006916676
C12	Verrucomicrobiota	0.002053388
C11	Actinobacteria	0.632442015
C11	Firmicutes	0.154675728
C11	Bacteroidetes	0.120845029
C11	Proteobacteria	0.069877382
C11	Other	0.019796129
C11	Verrucomicrobiota	0.002363717
C11	Fusobacteria	0
C10	Bacteroidetes	0.473175396
C10	Firmicutes	0.453096781
C10	Other	0.027515139
C10	Actinobacteria	0.025709126
C10	Proteobacteria	0.020397323
C10	Verrucomicrobiota	0.000106236
C10	Fusobacteria	0
C1	Actinobacteria	0.52406532
C1	Firmicutes	0.360012892
C1	Bacteroidetes	0.098839708
C1	Proteobacteria	0.016007735

C1	Verrucomicrobiota	0.00096691
C1	Other	0.000107434
C1	Fusobacteria	0

Supplementary Table S3: Differential abundance analysis results

Divergence	Comparison(s)	Genera
Increased abundance	pos_neg_common	Enterococcus; Sphingomonas; Staphylococcus
	pos_specific	Klebsiella; Parabacteroides; Proteus; Pseudomonas
Decreased abundance	pos_neg_common	Anaerostipes; Blautia; Collinsella; Dialister; Eubacterium; Faecalibacterium; Prevotella; Roseburia
	pos_specific	Bifidobacterium; Helicobacter; Lachnospira; Romboutsia; Turicibacter

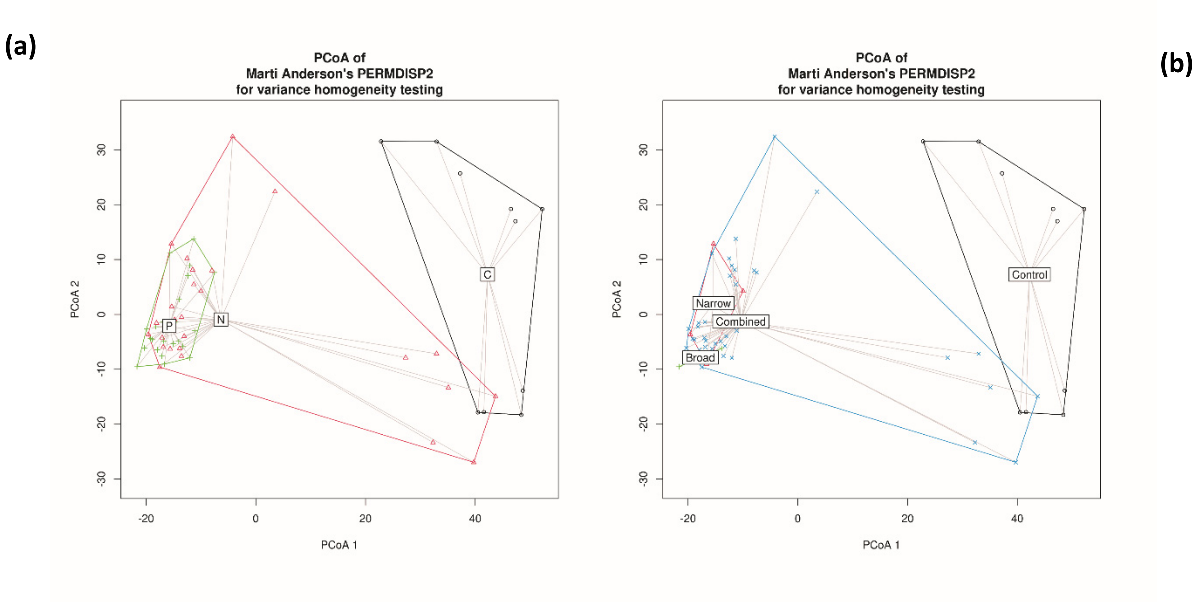

Supplementary Figure S1. Between groups variance heterogeneity for PERMANOVA evaluation. (a). Variance between CRE groups. Both ANOVA and permutation test suggest the existence of significant between groups variance of heterogeneity. Post-hoc Tukey HSD test located the difference between CRE-positive *vs* control and CRE-positive *vs* Cre-negative samples. (**b**)**.** Variance between antibiotic groups. Both ANOVA and permutation test suggest the absence of any significant between groups variance of heterogeneity. Adjusted or nominal p-value threshold of 0.05 was used to define statistical significance.

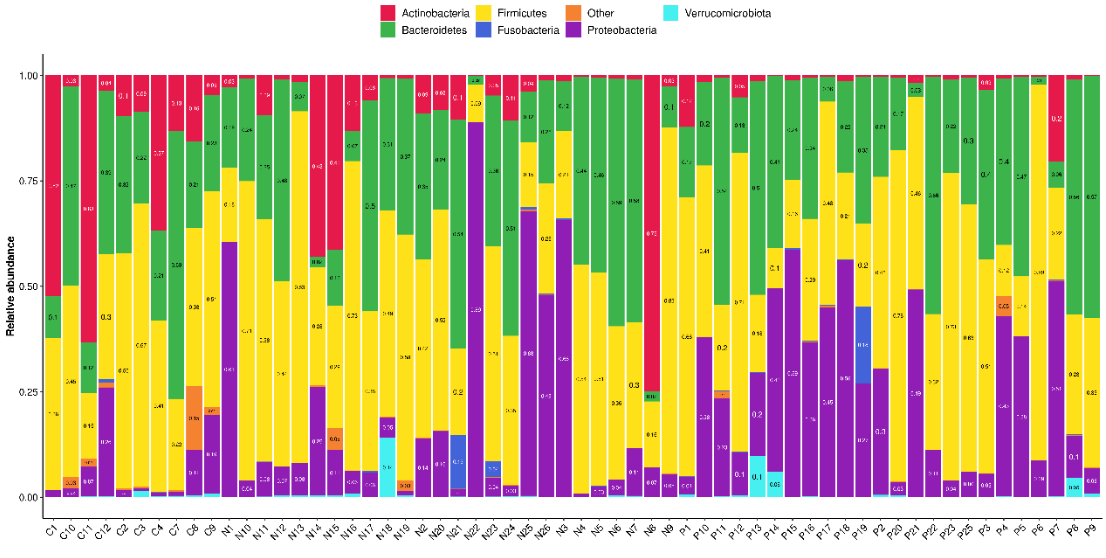

Supplementary Figure S2. Per sample top phyla relative abundance. All taxa having a gene copy number corrected relative abundance below the respective 3rd quartile were classified as “Other”.

(a)

(b)

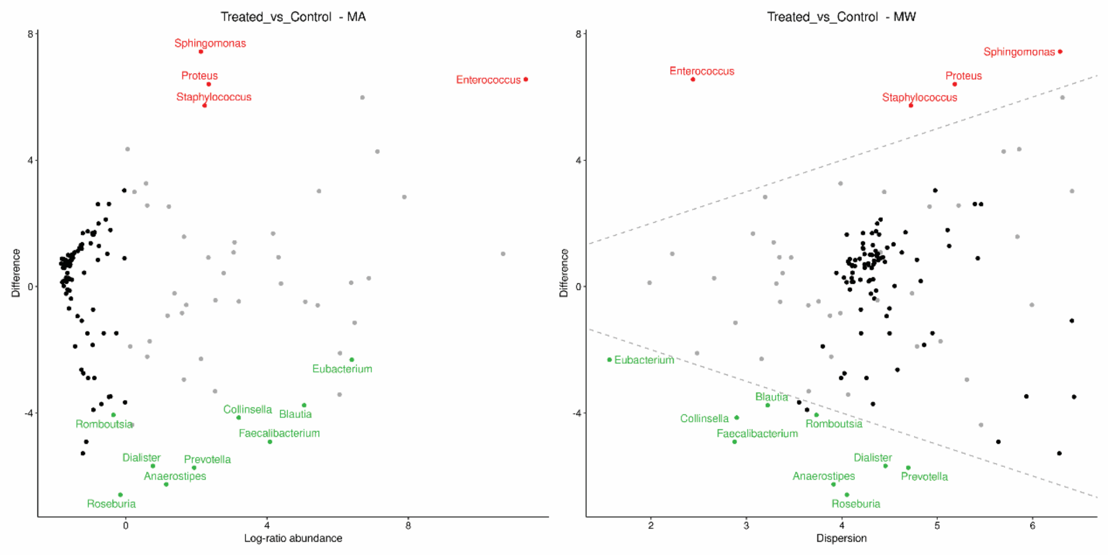

Supplementary Figure S3. Differentially abundant microbial genera between antibiotics treated and control individuals. (a) Bland-Altman plot. (b) Effect plot. Welch's t & Wilcoxon tests, as implemented in ALDEx2, were used to test for differential abundance. Absolute ALDEx2 effect size > 1 and adjusted p-value <= 0.05 were used as differential abundance thresholds. Genera found of increased abundance by both tests under treatment conditions are marked with red, while those found of decreased abundance are marked with green color.
